# The IARA Model as an Integrative Approach to Promote Autonomy in COPD Patients through Improvement of Self-Efficacy Beliefs and Illness Perception: A Mixed-Method Pilot Study

**DOI:** 10.3389/fpsyg.2017.01682

**Published:** 2017-10-05

**Authors:** Andrea De Giorgio, Angelo Dante, Valeria Cavioni, Anna M. Padovan, Desiree Rigonat, Francesca Iseppi, Giuseppina Graceffa, Francesca Gulotta

**Affiliations:** ^1^Psychology, Università Cattolica del Sacro Cuore, Milan, Italy; ^2^Kiara Association, Turin, Italy; ^3^Psychology, Università degli Studi eCampus, Novedrate, Italy; ^4^Department of Medicine, Surgery and Health Sciences, Nursing School, University of Trieste, Trieste, Italy; ^5^Department of Health, Life and Environmental Sciences, University of L'Aquila, L'Aquila, Italy; ^6^Brain and Behavioral Sciences, University of Pavia, Pavia, Italy; ^7^Engineering and Architecture, University of Trieste, Trieste, Italy

**Keywords:** IARA model, COPD, respiratory illness, guided imagery, educational health care, Awareness, autonomy

## Abstract

Chronic obstructive pulmonary disease (COPD) is one of the most deadly and costly chronic diseases in the world characterized by many breathing problems. The management of COPD and the prevention of exacerbations are a priority goals to improve the quality of life in patients affected by this illness. In addition, it is also crucial to improve the patients' adherence to care which, in turn, depends on their knowledge and understanding of some factors such as the prescribed medical treatment, changes in dailylife, and the process of breathing. In turn, the adherence to care leads to greater autonomy for the patient who is thus able to better manage his illness. Here we presented the application of the Model IARA in patients affected by COPD in order to achieve their autonomy in illness management which, in turn, leads to a better quality of life. IARA is an intervention program which improve the awareness and knowledge of patients with respect to both the disease and symptoms through health education. Moreover, through IARA the patients are encouraged to become more actively involved in COPD care process, also regarding drug therapy adherence. Using St. George's Respiratory Questionnaire combined with qualitative analysis, we demonstrated that IARA could be considered a useful approach in COPD management.

## Introduction

Chronic obstructive pulmonary disease (COPD) is one of the most deadly and costly chronic diseases in the world (NIH, 2007) characterized by breathing problems (Koblizek et al., 2016), in particular breathlessness and cough, which lead both to reduced physical activity (Corhay, 2015) and significant impairment of quality of life (QoL) with premature death (Koblizek et al., 2016).

The COPD acronym denotes a chronic inflammatory lung syndrome, primarily caused by long-term harmful inhalation, typically related to cigarette smoke which leads to progressive airflow limitation and bronchial obstruction (Buist et al., 2007; Gershon et al., 2011; Heijink et al., 2016). Drugs as bronchodilators are central to COPD management (Global Initiative for Chronic Obstructive Lung Disease, 2015) to prevent and control symptoms and to reduce frequency and severity of exacerbations, thus improving health status and resistance to exercise. The prevention of exacerbations is the priority goal in COPD management (Global Initiative for Chronic Obstructive Lung Disease, 2015). COPD requires long-term adherence to drug therapy and literature demonstrates how the level of adhesion by patients is usually very low, influencing therapy outcomes (Rogliani et al., 2017). In these patients the adherence to therapy enhances the QoL and education about illness through the so-called patient-centered care is very important (Epstein et al., 2010). In turn, adherence to therapy leads to autonomy by patients which improve the sense of self-efficacy and self-esteem (Bandura, 1997).

However, adherence and autonomy depend on not only by patients (e.g., patient's coping strategy and self-efficacy, health beliefs, psychological profile), but also by physicians (e.g., method of administration and dosing regimen also taking into account the patient's needs), nurses (e.g., quality of nurse care), and society (e.g., quantity and quality of caregivers, social support).

In order to improve the QoL in patients affected by this disease, scientists have suggested different adjuvant to medical treatment, such as psychological (for anxiety and depression) and psychosocial support (Farver-Vestergaard et al., 2015), respiratory motor training (Ovechkin et al., 2016), physical activity (Araújo, 2016) mindfulness-based (Mularski et al., 2009), and educational interventions (Harris et al., 2008) also for healthcare professionals (Pelland et al., 2017). However, taken individually, these approaches have not always given satisfying results.

We describe hereunder four cases where an integrative approach named IARA (Padovan, 2014) was carried out. IARA is an italian acronym (Incontro, Alleanza, Responsabilità, Autonomia) which means Meeting, Compliance, Responsibility, Autonomy and its main purpose is making autonomous the patient in her/his unique illness experience.

This model was already used on chronic tension-type headache (Gulotta et al., 2015) and gastroesophageal reflux (De Giorgio et al., 2017) with encouraging results particularly toward emotion management (De Giorgio, 2016).

IARA is based on Assagioli psychosynthesis, a psychological theory for the conscious attempt of the humans to cooperate with the natural process of personal development, also through practical techniques such as meditation, guided imagery, and the use of evocative words (Assagioli, 1965).

Transpersonal psychology is also part of this model (Grof, 1975) and spiritual approach is considered a second generation of mindfulness-based interventions because it takes account of the whole person (Van Gordon et al., 2015), including the natural tendency to the transcendent, not necessarily meant as a religious concept (Schaub, 2013).

Here we demonstrated as IARA could be considered a useful approach in COPD management because leads the patient to a state of autonomy in order to deal with his/her own situation and to give a different meaning to the illness experience, it allows to achieve an improved self-awareness and proper knowledge about the personal therapy, improving an active and responsible participation in the care process.

## Materials and methods

In order to improve the QoL in patients affected by COPD, IARA model has used the following tools: (I) patients education about his disease; (II) a guided imagery meditation (GI) called “*Love and light breath*” (Box 1) that patient performed on a daily basis; (III) the use of anatomical interactive model of respiratory structures (Table 1); and (IV) drawing by patients about illness perception (Table 1).

Box 1 **Guided imagery meditation: “love and light breath” exercise.**

*Let's put ourselves in a comfortable position, let's pay attention to what is happening within ourselves through our breathing, let's connect us with our breath rhythm without speeding it up or slowing it down*.

*Now while breathing let's imagine to carry within us a calm, bright and loving breath. With the air coming out, leave all those things that are no longer useful in this moment*.

*Through our breath let's get in contact with our physical level, bringing the bright and loving air to the bottom of the body, touching our feet, legs, and pelvis. Let's imagine our cells feeding on that fresh, bright, and loving air, freeing themselves from what they have accumulated. With the air coming out, leave all those things that are no longer useful in this moment*.

*Let's continue keeping in touch with the breath, and with all senses, and let's imagine that our cells are nourishing themselves of fresh, bright, and loving air, in this way our cells can be free from what they have accumulated. With the air coming out, leave all those things that are no longer useful in this very moment*.

*Now through our breath let's get in touch with our emotional part, let's imagine a calm and clear lake, reflecting the surrounding landscape illuminated by a radiant sun*.

*Let's imagine a fresh, bright, and loving air blow stirring our emotions and, with the air coming out, let's imagine that all those emotions that, in this moment, are no longer useful, are going out*.

*Now, through our breath again, let's get in touch with our mental level, let's imagine a room with windows. Let's open these windows and let a bright and loving air enter, running through our thoughts. From the windows we can see the air going out making us free from those thoughts that are no longer useful, creating a new space that allows us to have new thoughts, and new insights*.

*Let's continue breathing*.

*Now with the breath let's move ourselves to the center of our heart, where we can imagine to switch on a light. Let's move this light, such as a lighthouse or lamp, to the body area asking for help and care*.

*We can see that both of our lungs light up: the trachea, both the main bronchus, the lobar bronchus, the segmental bronchus, the conducting bronchiole, the terminal bronchiole, the respiratory bronchiole, and all the air sacs*.

*Let's imagine some little men leaving from the center of the heart, as a group, with a joyful attitude, like quiet and cooperating workers that are going along the lighted way. We can see that these men are divided into subgroups and are distributed in various areas: they start from the trachea, then they go down through bronchus, the lobar bronchus, the segmental bronchus, the conducting bronchiole, the terminal bronchiole, the respiratory bronchiole, and arrive into all the air sacs. We can see how the little men keep open and co-operate to the functionality of the air sacs*.

*Let's remain a moment to observe and look at what is happening, how they work, if we receive images, feelings, thoughts, colors, insights that are useful for this particular moment. Let's continue breathing emitting light and loving air that facilitate the men's work. With the air coming out let what is no longer useful for now go away*.

*When we see that the men have finished their task, always breathing, we can see them retiring and move toward the center of the heart bunching up the initial group with a joyful, cooperative, and compassionate attitude. Now, through our breath, let's imagine spreading the gratitude to our physical, emotional, and mental levels, in order to be grateful for the experience that we have lived and for them taking care of us. With the air coming out, let what is still present, but no longer useful, go away. Now let's bring the attention to the surrounding environment, let's get in touch with the outside world and bring the attention to the awareness. Accelerating our breath, let's start to move our hands, our feet and, when we are ready, open our eyes*.

**Table 1:** Drawing of illness perceptions and solutions and education about respiratory system.

	**The respiratory system**	**The illness**	**Solution**
Case 1	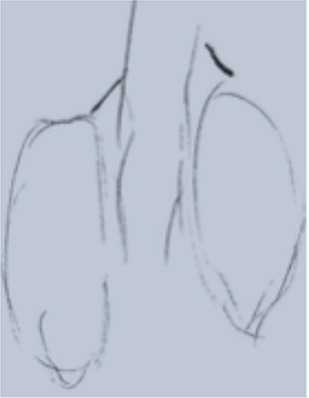	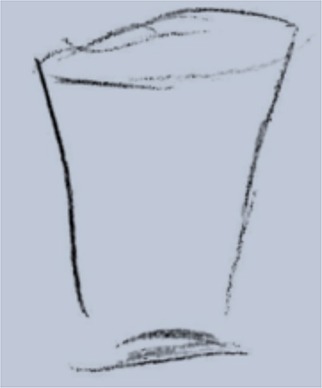	The illness is perceived as a cork, and can be solved by removing it.
Case 2	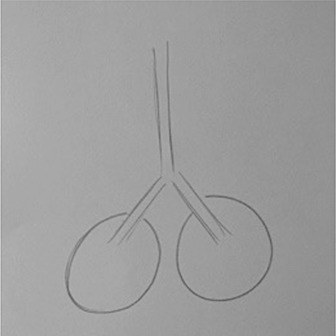	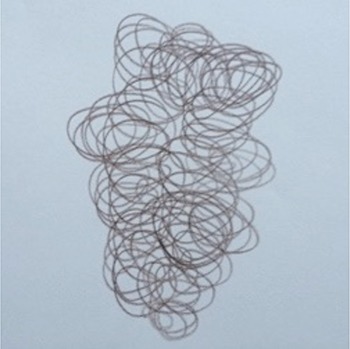	The illness is perceived as a tangled mass, and can be resolved by unraveling it and making it lighter.
Case 3	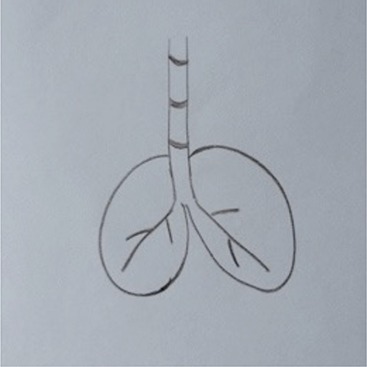	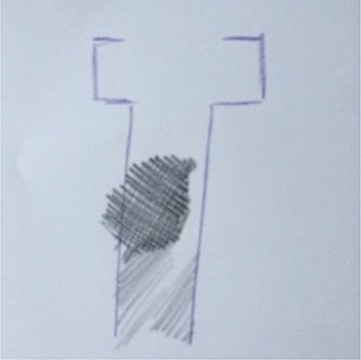	The illness is perceived as a clogged chimney. In order to solve it a chimneysweeper is needed. Furthermore, by reducing cigarette smoke, the soot accumulated in the chimney would also be reduced.
Case 4	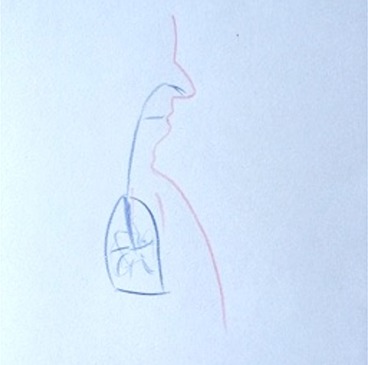	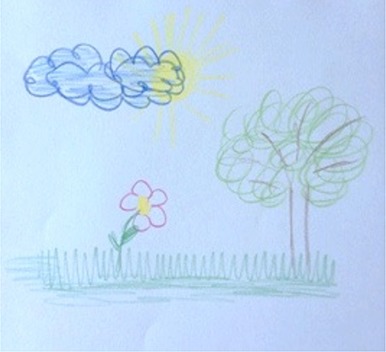	The illness is perceived as a cloud that casts a shadow on the patient's life, represented by a meadow with flowers and trees. The solution to the illness is to turn the clouds away to remove the shadow.
Anatomical interactive model of respiratory structures	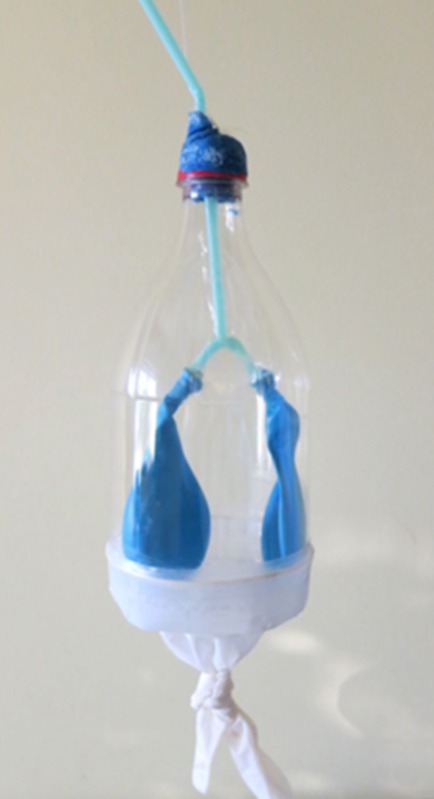	In this picture it is represented the model of the respiratory system used to educate patients about its anatomy and physiology.	

*Here it is possible to observe the process of patients' illness awareness. Starting from their disease knowledge about respiratory system it is asked to draw how they imagine their illness. Then, it is asked to patients to imagine which solution they would adopt to recover symptoms. Finally, it is explain the structure of respiratory system through the use of anatomical interactive model*.

The program (Box 2) was carried out between the months of July and September 2016 and was conducted and structured in four meetings by a nurse with a Master's Degree in Psychology (from now-on referred to “nurse”).

Box 2 **Weekly meetings.**

First meeting (T0): the nurse has met the patients, creating a positive and judgement-free listening environment according to Carl Rogers' theory in order to establish a relationship of mutual trust and alliance. Patients were put at ease and let talking freely about their lives and illness. Then, the patient performed the treadmill exercise.

The main purpose of the second meeting (T1; 7 days from the first) was to raise body and disease patient awareness through the drawing. To this end, the nurse provided the patients with blank sheets and a box of colors, asking them to draw what they knew about the anatomy and physiology of the respiratory system. Furthermore, in order to provide the patients with full awareness of the respiratory dynamics and the physiopathological alterations caused by COPD, a model called “the breathing bottle” was introduced (see Anatomical interactive model of respiratory structures; Table 1). The patients were asked to observe it and study its functioning, to visually understand what happens in case the endings of the straw are reduced in diameter. By gently pressing the straw the patients understood how lung expansion and volume are reduced. The patients' inquiries about the illness were then addressed and, following this, they were again asked to represent through a drawing how the respiratory problem was perceived and what could be an effective solution. Based on the drawings, the therapeutic education was completed in regard to the usage of drugs and the importance of performing respiratory exercises on a regular basis.

The third meeting (T2; 14 days from the first) focuses on guided imagery meditation, through the exercise called “love and light breath.” This exercise was proposed to the participants to gain full awareness and perception of their own bodies. The guided imagery meditation exercise is structured in two parts: the first is a preparatory phase for aligning the physical, mental and emotional layers by means of specific visualizations and terms; the second is a proper guided imagery meditation moment where the image of “the little men at work” is introduced. These types of images favor a playful and relaxing attitude while performing the exercise and encourage the process of disidentification between person and illness. Moreover, aim of this meeting is unleash the patients' potential to re-establish equilibrium in their health status through self-efficacy. To this end the following approaches were proposed to identify qualities by choosing “evoking words.” Regarding the “evoking words,” the patients were invited to write down seven qualities that they considered to have and, discussing on these seven, pick two to use them during the entire rehabilitation process. These two words, in particular, help the patient to face the entire rehabilitation process. The patient can add new own qualities during the month and choose the two qualities that he/she feels most useful in a particular period.

Finally, in the fourth meeting (T3; 21 days from the first), the nurse proposed again the usage of the evoking words and guided imagery meditation in order to evaluate the effective adhesion of the participants. During the time span across the meetings it was in fact asked to the patients to repeat at home the exercise with guided imagery meditation and the evoking words.

To evaluate the influence of the illness on the health status of the participants we used the St. George's Respiratory Questionnaire (SGRQ; Meguro et al., 2006) administered before the first IARA meeting and after 3 months from the last meeting. SGRQ is structured in three components scores: symptoms, activities, impact of respiratory illness. The first one measures the effects of the respiratory symptoms, the frequency and severity over a preceding period that may range 1 month to 1 year. Moreover, its purpose is to assess the patients' perception of their recent respiratory problems.

The second one covers both the patients' current state (i.e., how they are these days), measures disturbances to patients daily physical activity. The last one measures the impacts of illness, a range of disturbances of psycho-social function. This component relates both to respiratory symptoms and also correlates with exercise performance (i.e., as 6-min walking test), disturbances of mood such as anxiety and depression, a breathlessness in daily life. Scores of individual evaluation areas and total scores were calculated from the last measurement performed during the study. The questionnaire response has a unique empirically derived percentage weight, ranging from 0 to 100. The closest percentage is to 100, the worse is the health condition of the patient. A change of at least 4 units is considered the minimum variation clinically significant by the patient (Jones, 2002).

Prior to data collection, a written consent was submitted and obtained from the patients. This study was carried out in accordance with the recommendations of “name of guidelines, name of committee” with written informed consent from all subjects. All subjects gave written informed consent to participate in the study in accordance with the Declaration of Helsinki and Good Clinical Practice. Moreover, each patient gave his/her consent for the publication of this case presentations. Finally, the protocol was approved by the Ethics Committee of the eCampus University.

## Case presentations and results

### Case 1

The first participant was a 63-year-old female with a moderate COPD, diagnosed according to stage II GOLD classification criteria (FEV_1_/FVC <0.70 – FEV_1_ 50–79% normal). The anamnesis has revealed that the patient usually smokes (30 cigarettes/day) as single behavioral risk factor. We detected also physical exertion intolerance, dyspnea, and productive cough, especially in the morning. The patient declared limitations on Activities of Daily Living (ADLs), such as bathing, housekeeping, and walking. The specific patient's therapy was Tiotropium (Spiriva®) 18 mcg/1 inhalation per day.

### Case 2

The second participant was a 75-year-old female with a severe COPD, diagnosed according to stage III GOLD classification criteria (FEV1/FVC <0.70 – FEV1 30–49% normal). The anamnesis has revealed that the patient usually smokes (10 cigarettes/day). In the past she used to work as a tailor and prior to the illness she used to be very active (e.g., she used to dance with her husband). She states that nowadays she is not able to carry out housekeeping chores adequately and is not able to endure physical effort any more. The specific patient's therapy was Tiotropium (Spiriva®) 18 mcg/1 inhalation per day, Fluticasone fuorate/Vilanterol (Revinty®; Ellipta®) 92/22 mcg/1 inhalation per day and Oxygen Therapy 2 L/m for 12 h/day.

### Case 3

The third participant was a 75-year-old male with a moderate COPD, diagnosed according to stage II GOLD classification criteria (FEV_1_/FVC <0.70 – FEV_1_ 50–79% normal). The patient used to smoke 7 cigarettes per day. He is a former football player and prior to the illness flared-up, he used to have an active social life, compatible with his respiratory functionality. He does not declare relevant alterations in ADL, but states concerns about his future. The specific patient's therapy was Tiotropium (Spiriva®) 18 mcg/1 inhalation per day. He showed good adherence to the treatment plan.

### Case 4

The fourth participant was a 63-year-old female with a moderate COPD, diagnosed according to stage II GOLD classification criteria (FEV1/FVC <0.70 – FEV1 50–79% normal). The woman is a former smoker and currently reports difficulties in performing housekeeping chores and activities that require any kind of physical effort. The specific patient's therapy was Tiotropium (Spiriva®) 18 mcg 1 inhalation/day.

Here we analyzed four patients, three of them with moderate COPD and one with severe COPD. No alteration was made to four patients pharmacological treatment during the period of the study, but each patient declared more adherence to the treatment plan.

After the first meeting that was useful to establish a relationship of mutual trust and alliance, the IARA model pointed out to increase the body awareness through the use both the drawing (second meeting; Table 1) and anatomical education through a scale model of respiratory system (Table 1). The achievement was confirmed in patients' experiences (second meeting; Table 2) which increases with following meetings.

**Table 2:** The experience of the patients.

	**Meeting 1**	**Meeting 2**	**Meeting 3**	**Meeting 4**
Case 1	I would never have imagined that such a thing could happen to me. It is not really clear to me what it is, but my life now is not as good as it used to be: I am not free to chase after my grandaugther and bathing and getting dressed now require a big effort, since I cough mostly during the morning hours. Generally I am not able to expectorate fully and effectively. It is unnerving not being able to climb the road to home: I always have to stop halfway to catch my breath.	I thought it [the respiratory system] worked the other way around; this means that everything that raises the diaphragm or prevents it from lowering down will not be effective for my breathing. Thanks, it has been useful to see how it works, and I had a lot of fun. So, if I eat foods that swollen the abdomen the diaphragm raises and my breathing gets worse. Now I understand why I did not feel well when I ate too much. Muscles help in breathing and thus it is good to training them. Even if working out or carrying out breathing exercises is difficult, it is important to do them to avoid being even more fatigued in the future. In addition, I had the opportunity to gain awareness of my illness. When I feel I cannot breathe it is like someone was putting a cork down my throat that does not let air pass through; it is like a chimney that when it is clogged does not allow the smoke and smells to get out. Then, maybe I should quit smoking. If smoking makes the cork grow, maybe quitting could help me delay the call to the chimneysweepers. It is a matter of diameter in the end: I either have to remove the cork, or to enlarge the chimney.	Listening, helping others, not judging, love for beauty, precision, determination, courage.	It has been relaxing; in my mind the little men were like Snow-white's dwarfs helping me. I found the image of the lake very beautiful; it gave me a sense of serenity and tranquility. I would sit in front of my window thinking I was on the sea shore and do the breathing exercises feeling free and relaxed: it was a moment just for myself. Every time I would inhale the drugs I thought of the respiratory system and the little men carrying the particles around. It was like I could feel myself expanding.
Case 2	I hate this illness. Before I used to do everything on my own, nowadays my husband has to do everything for me. It is a pain. I can't go dancing anymore, nor going out as I did before. If I go out twice per day now, it is already a lot. I need 40 min to climb 4 flights of stairs, so I often prefer to stay at home. Furthermore, having to go out with this [oxygen] can is a pain: everybody stares at you, talk behind your back and look at you with pitiful eyes. Even if I wanted to move around the house, it still weights a ton. I detest being like this.	So, lungs are expanding and the diaphragm rise up, I thought it was far more complex, and that the movements were the other way around. They were right indeed to tell me to quit smoking. I never did it because I found it pleasing and did not understand why I should quit. Now I understand but I don't believe I will quit smoking. I will try to smoke less at least.	Energy, activity, volcano, precise, obstinate, punctual, exigent.	Interesting, different, seemed like a fairy tale. In the little men I saw the drug going toward its destination. I liked this program, I know I will not heal but I will try my best to delay the worse. Thanks.
Case 3	It is not clear to me what it [the disease] is, I even did not know the meaning of the acronym. I have difficulties in breathing. It seems it is called dyspnea, but I do not know why. I quit playing football with friends years ago. Every once in a while I would like to go walking in the mountains with them, but I can't. It makes me uncomfortable. Who knows what will become of me.	There is nothing I can do about the smog and other harmful fumes, but at least I could limit the cigarettes. The chimney will not get cleaner but at least it will also not get dirtier. You know, it is a bit like back in the times when I used to play football: if we knew the strategy of the opponent in advance, it was much easier to get prepared and face the match. You knew what you could expect and you knew how you could overcome difficulties. Hence, if I use the bronchodilator and the cortisone I can prevent my chimney/pipe to get completely clogged.	Cheerful, determined, understanding, playful, exigent, gritty, strong-willed.	The little men are like my football team. That's how I imagined them, competitive and determined. This makes me smile, it is fun, but is also helpful to get in touch with your body, to see it in a way that is familiar to you. It is also useful for drug administration. Thanks for this experience and for helping me. Now I am the coach!
Case 4	I am not able to carry out housekeeping chores any more, I struggle to lift my grandson, I depend on my husband for many works and activities like carrying the grocery bags. Furthermore, I really don't understand why in the morning I have all this cough and secretions. It really puts me off, but I have to stay at home until it passes.	So, if all these little tubes are clogged, there is not much they can do. That's why I can't breathe or I do it with a lot of difficulties. But if I keep exercising maybe I can prevent them to get entirely obstructed. Having tried out the devices in peacefullness and with you explaining everything to me makes me much more relaxed. It was useful also to learn what all these medicines are for.	Love for others, altruism, carefreeness, determination, cheerfulness, jolliness, creativity.	Doing this at home is very relaxing. At times I think of the little men and this makes me smile and breathe more deeply. It has been useful to be trained in breathing.

In particular, in Table 1, under “*The illness*” it is possible to observe how all patients draw a respiratory efficiency reduction (a cork, a tangled mass, a clogged chimney, and a cloud), each one with his particular perception.

### Data analysis

Paired-samples *t*-test was used to analyze the differences between pre- and post- conditions in the SGRQ dimensions for all patients and to compare pre and post daily cigarettes' consumption for case no. 1, 2, and 3.

**Table:** 

	**SGRQ Dimensions**							
	**Symptoms**		**Activities**		**Impact**		**Total**	
	**Pre**	**Post**	**Pre**	**Post**	**Pre**	**Post**	**Pre**	**Post**
Case 1	88.8	90.7	92.5	79.1	72.5	57.6	81.2	69.6
Case 2	50.8	50.4	35.7	35.7	18.1	11.1	28.4	24.5
Case 3	72.4	86.3	92.5	92.4	87.4	61.5	89.6	75.0
Case 4	81.9	81.9	79.8	66.3	47.3	29.2	62.5	48.4

Results showed that participants experienced significantly lower scores in the dimensions related to the social and psychological impact of the illness at post-test (*M* = 39.85) compared with the pre-test condition (*M* = 56.32), *t*_(3)_ = 4.2, *p* = 0.024.

Furthermore, on average a significant decrease was found in the total sum of the SGRQ dimensions comparing the pre-test (*M* = 65.42) with the post-test condition (*M* = 54.37), *t*_(3)_ = 4.47, *p* = 0.021.

Despite the difference was not significant *t*_(3)_ = 1.75, *p* > 0.05 the participants reported lower levels of activities during the post-test (*M* = 68.37) compared with the pre-test phase (*M* = 75.12). A decrease in daily cigarettes' consumption was observed, however difference between pre (*M* = 15.66) and post-phase (*M* = 7.33) was not significant, *t*_(2)_ = 2.92, *p* > 0.05.

## Discussion

It has been demonstrated in COPD that non-pharmacological interventions such as exercise, patient education, breathing strategies, and mind-body interventions are important methods in addition to pharmacological treatment (Guthrie et al., 2001; Bausewein et al., 2008). In particular, regarding mind-body interventions in patients with COPD such as mindfulness and GI, it has been proven their effectiveness (Benzo, 2013; Chan et al., 2015; Jones, 2015) in term of relaxation, stress reduction, enabling a less distressing interpretation of physical symptoms (Mularski et al., 2009). These interventions can be considered as a receptive awareness and registration of inner experiences and external events (Bishop et al., 2004). It has also been shown that mindfulness meditation training raises awareness of the interoceptive signals coming from the body (Farb et al., 2013) reducing anxiety, distress and even depression (Goldin and Gross, 2010; Gallego et al., 2015; Zhang et al., 2015; Crowe et al., 2016) improving the QoL (Grazzi et al., 2017). It also has been demonstrated both this mind-body shift increases the interoceptive attention awareness (Critchley et al., 2004; Farb et al., 2013; Kerr et al., 2013) and GI exercise has neural foundations (Kosslyn et al., 2001) useful also in emotional disorders (Holmes and Mathews, 2010), in management of symptoms in patients receiving chemotherapy (Charalambous et al., 2016), and among patients with inflammatory bowel disease (Mizrahi et al., 2012; De Giorgio et al., 2017).

Furthermore, in order to ameliorate social and economical status in people affected by COPD the psychological approach should not be underestimated. Johnson et al. (2007) reviewed the literature of COPD regarding social consequences caused by this disease. In particular, the authors explored the relationships between COPD and stigma and gender, focusing on how elements such as cultural norms and values may interact to affect experiences of these patients within their social environment. The researchers argue that the stigma of COPD arises because people affected by this illness are held responsible for their conditions, particularly because of smoking which is considered inappropriate behavior. These people are extremely recognizable because of their bodily changes (considering also physical restrictions) and oxygen equipment, which can lead both to reduced community mobility and to increased social exclusion, also in their own working context.

The IARA model that we have presented takes into account all aforementioned approaches, making an effective synthesis of these aspects. In our work we proposed the “*Love and light breath”* exercise created ad hoc for COPD. According with literature (Lahmann et al., 2010), this type of exercise could bring the participants to gain full awareness and perception of their own bodies.

Also GI meditation exercise adopted in the IARA model lead to an increase of body consciousness, because the “*men used*” during visualization allow to shift the attention from mind to signals coming from the body (see Kabat-Zinn, 2005; Hölzel et al., 2011). In particular, the GI exercise and education used in IARA let the patient to rise awareness about drugs and their effect into the body. Therefore, drugs become substances the patient learn to know and accept increasing the adherence to care.

Patients confirming their positive experience with GI exercise as highlight in fourth meeting in which they described the exercise such as “*relaxing”; “sense of serenity and calmness,” “interesting, different, seemed like a fairy tale”; “useful for drug administration,” “breathe more deeply.”*

IARA stimulates also the patient to reflect his fundamental qualities (see third meeting; Table 2), and these qualities, people are often not aware of, lead to a conscious involvement in the care process. This conscious involvement, in turn, stimulates both responsibility and autonomy, increasing the therapy and exercise adherence.

Awareness and acceptance of illness are highlighted, for example, in case 2 (Table 2) where the patient, during the first meeting, claims “*I hate this illness*,” while in the fourth meeting claims “*I liked this program, I know I will not heal but I will try my best to delay the worse. Thanks*.”

In the cases we are presenting, the cited evidences are in agreement with an improvement of respiratory symptoms: patients may have ameliorate their symptom perceptions which, in turn, lead to a better management of illness. In this way it is also possible to explain the diminishing in frequency, because patients may perceive their symptoms less burdensome, not including them in SGRQ (see also the next paragraph “*The reasons why can be useful to implement the IARA model in COPD care plans*”).

Due to the COPD, patients have several changes in life-style. These changes impair the QoL and, consequently, lead to a diminished patient's role within the family and community. Several studies have also highlighted that psychological distress predicts restricted activities of daily living and, in particular, when this is associated with dyspnea affects the level of physical activity (Beck et al., 1988; Weaver and Narsavage, 1992; Graydon and Ross, 1995). It has also been shown (Carter et al., 1988) that pulmonary rehabilitation is able to ameliorate the QoL in patients with COPD because it helps them to increase their activity achieving a higher level of functioning and independence.

Our study documented an improvement in health-related QoL in the patients considered, confirmed by a decrease in the overall score of the SGQR. Our results can be comparable with those observed in other studies (Goldstein et al., 1994; Wykstra et al., 1994; Paz-Díaz et al., 2007) and we hypothesize that through IARA model patients have increased their adherence to care in four meetings. Benzo (2013) has already discussed as mindfulness and motivational interviewing may be useful to promote self-management in COPD, but these interventions during, respectively, 8-week and 3 months but do not take into account patient's educational care and full awareness of his pathology.

Indeed, in COPD it is also very important to note the patient's self-efficiency, because this belief is known to increase the person's motivation, perseverance, thought patterns, course of action, emotional response, and attribution of accomplishment and failure (Bandura, 1997). In order to improve the patient's self-efficiency in this type of pathology is crucial the education to the disease alongside exercise programs (Zimmerman et al., 1996; Scherer et al., 1998). According to that, in the second meeting we used “the breathing bottle” in order to educate the patient through the explanation about respiratory system (see Table 1).

Aim of third meeting was unleash the patients' potential to re-establish equilibrium in their health status through self-efficacy. To this end were proposed to identify qualities by choosing *evoking words* according to Psychosynthesis principles. In this way patients have been able to go through themselves qualities, improving the self-efficacy as argued by themselves in fourth meeting.

Finally IARA is an approach that responds to basic principles of patient-centered care (Epstein and Street, 2011). The person affected by COPD, as well as any illness, has values, preferences, and desired health outcomes related to her/his unique experiences, background, and on her/his thoughts in regard of QoL. The patient-centered care approach is based on different relationship between healthcare professionals and patients and, therefore, is a quality of personal, professional, and organizational relationships.

In particular, a care provider does not prescribe the same treatment for most patients affected by similar conditions, but takes into account the patient's unique concerns, preferences, and values, focusing on the health outcomes that are important to each patient. Finally, it may be useful to emphasize that patient-centered care does not mean that hospitals adopt models used by boutique hotels with greeters, gadgetry, and greenery, because these things do not necessarily contribute to achieve the patient-centered objectives (Epstein and Street, 2011).

## Conclusion

Many studies (see for example, Martin et al., 2005) agree that the improve of patients adherence to care and autonomy depend on their knowledge and understanding of some factors such as the prescribed medical treatment and changes in daily life (diet, smoking cessation, physical activity etc.). It is also very important the nurturance of trust in the therapeutic relationship and effective communication among health professionals, patients, and *caregivers*. Even though this is theoretically known, it has been discussed that ~50% of the patients with a chronic disease have further problems following forgetting, misunderstanding, and ignoring healthcare advices which lead to scarce clinical benefit (Dunbar-Jacob et al., 2000). Then, the patient's full understanding of COPD disease (symptoms, limitations, residual functionality) is crucial to achieve a better therapeutic compliance.

The IARA model is useful because it has been shown that single intervention strategy cannot improve the patient's adherence to care and autonomy in management of illness (Epstein and Street, 2011). Indeed, as already discussed by Martin et al. (2005), patients need to have the opportunity to tell the story of their unique illness experiences and, knowing them as a person, allows the health professional to understand decisive elements in order to achieve the patient's autonomy such as their beliefs, subjective norms, attitudes, and emotional health challenges (anxiety, depression, distress). In addition, the close cooperation among healthcare professionals, patients, and *caregivers* lead to a greater satisfaction, reduces the risks of non-adherence, and improves patients' QoL.

IARA has also the purpose of transforming a healthcare professionals in order to be more mindful, informative, and empathetic, moving their role from one characterized by authority to one characterized by collaboration with patients and *caregivers*.

Therefore, IARA could be considered an innovative educational and psychological model of care alongside pharmacological treatments because, as affirmed by literature (Hibbard, 2004; Epstein and Street, 2011), it is necessary moving toward a more patient-centered health care system. Indeed, the IARA model takes into account both the role of patients in their individual experience of illness and give to healthcare professionals several tools useful to establish partnership, solidarity, empathy, and collaboration among their and patients.

## Limitations of the study

We are aware that the described cases are not generalizable and statistically relevant data due to sample, but the results obtained by individual patients on the management of symptoms, the impact of the disease on ADL and the QoL, have considerable clinical significance, also for the smoking reduction (Melillo and Melillo, 2004). In particular, the use of drawing according to IARA model in the smoking reduction may have played a crucial role growing the awareness of the smoking effects. Due to IARA model, patients have empirically reduced the smoked cigarettes and this is a very important result because the level of cigarette exposure reduces the levels of carbon monoxide exhaled, determines positive effects on inflammatory processes of the bronchial mucosa and reduces functional decline, improving the QoL expectancy in people affected by COPD (Tønnesen et al., 2007).

Our result is consistent with what is documented in literature when patients follow a smoke reduction program (Tønnesen et al., 2007) and it is known that the reduction of cigarettes number promotes the subsequent termination (Wennike et al., 2003). The sample extension could lead to a statistically significant data about smoking reduction.

Therefore, quantitative and qualitative studies are necessary to show the full effectiveness of the IARA model in COPD, but the results of these cases are extremely encouraging.

## Author contributions

ADG and AP designed research; ADG, DR, AD, GG, FI, FG, AP performed research; VC and AD analyzed data; ADG, AD, GG, FG wrote the paper.

### Conflict of interest statement

The authors declare that the research was conducted in the absence of any commercial or financial relationships that could be construed as a potential conflict of interest. The handling Editor declared a shared affiliation, though no other collaboration, with one of the authors ADG, and the handling Editor states that the process met the standards of a fair and objective review.
